# Case of Arsenical Poisoning

**Published:** 1860-05

**Authors:** G. S. Fouke


### CASE OF ARSENICAL POISONING.

REPORTED BY G. S. FOUKE.

Miss M. E. S——n, a petite and lovely young lady, in her seventeenth year, came to us at Westminster, Md., on the 11th of February, 1860, to see what could be done for two of her teeth,—the upper right central and lateral incisors.

A dentist, from Pennsylvania, being in her neighborhood, (Wakefield, Carroll county, Md.,) driving the important business of "fixing teeth," Miss S. was induced to employ him to plug the teeth named above.

Miss S. possesses good teeth—these two being the only ones decayed. The central incisor was plugged on the post-approximal, and the lateral on the ante-approximal side. Inflammation supervened soon after the operation, and the face swelled to a great extent. Dr. Cooke, the family physician, being called, succeeded in subduing the alarming symptoms; but the teeth and gums continued in a very unpleasant condition. Over four weeks after the teeth were filled, Miss S. came to see us. We found the gum in front very much thickened, puffy, and of a dark or livid hue. From an unhealed wound, pus was oozing quite freely. The teeth, very loose, and projecting from the sockets considerably. The probe showed that the entire alveolar process was obliterated; and that there was, at the anterior base of the process, an exfoliation, extending from the canine fang to the extremity of the fang of the left central.

The injury was now irreparable. The teeth being processless, and the existence of an exfoliation at the extremity of the roots, rendered the removal of the teeth necessary.

The plugs were removed, simply to see the condition of the cavities. The central incisor was decayed only *epidentinally.* The lateral was decayed *subdentinally*, the pulp cavity exposed at a small fissure-like opening. The pulp cavity contained the remains of the deadened pulp.

In our examination of the case, the idea struck us that

*arsenic* must have had something to do in the production of this extensive destruction. We inquired of Miss S., whether she had any knowledge of what the dentist did to her teeth preparatory to plugging? She immediately remarked that the dentist put in her teeth, the day before he filled them, "rats-bane mixed with some oil of cinnamon." This satisfied us that arsenic had had a good deal to do in the matter. We removed the teeth, syringed the wound with *glycerine*, and gave Miss S. a wash to use, when she left us. We have reason to hope the mouth will heal, but we anticipate an ugly depression, from the loss of the gum and process.

We regard Dr. J. D. White's preparation of arsenious acid, morphia, and creosote, a very good remedy for destroying the dental pulp; we have used it for many years, and have experienced no injurious effects from it. *Crude* arsenic, in some *savory* oil, is not the way to use the article. Is it not a matter of surprise that any practitioner, now-a-days, should use the dangerous drug in this uncertain, unsafe form?

Prof. White's recipe we would append, as a suitable conclusion to the above:

Arsenious acid, xx grs.
Sulphate Morphine, xxx grs.
Creosote,—sufficient to make a paste.

The arsenious acid and morphia ground into the finest powder, before adding the creosote. A small speck, of the size of a pin's head, if laid on an exposed nerve, and properly secured *in situ*, will generally destroy a pulp in from 12 to 24 hours.

It is now five years since we prepared what we are now using, and we find *age* improves the efficiency of the mixture. The above proportions are given from memory, if they are not correct, will the Editors of the *Register* please state them correctly. We should not ask this, but we are away from home, and have no chance of referring to books.

